# High‐Throughput Approaches to Engineer Fluorescent Nanosensors

**DOI:** 10.1002/adma.202411067

**Published:** 2024-11-12

**Authors:** Justus T. Metternich, Sujit K. Patjoshi, Tanuja Kistwal, Sebastian Kruss

**Affiliations:** ^1^ Fraunhofer Institute for Microelectronic Circuits and Systems Finkenstrasse 61 47057 Duisburg Germany; ^2^ Department of Chemistry Ruhr‐University Bochum Universitätsstrasse 150 44801 Bochum Germany; ^3^ Center for Nanointegration Duisburg‐Essen (CENIDE) Carl‐Benz‐Strasse 199 47057 Duisburg Germany

**Keywords:** biosensors, carbon nanotubes, fluorescence, high throughput, nanomaterials

## Abstract

Optical sensors are powerful tools to identify and image (biological) molecules. Because of their optoelectronic properties, nanomaterials are often used as building blocks. To transduce the chemical interaction with the analyte into an optical signal, the interplay between surface chemistry and nanomaterial photophysics has to be optimized. Understanding these aspects promises major opportunities for tailored sensors with optimal performance. However, this requires methods to create and explore the many chemical permutations. Indeed, many current approaches are limited in throughput. This affects the chemical design space that can be studied, the application of machine learning approaches as well as fundamental mechanistic understanding. Here, an overview of selection‐limited and synthesis‐limited approaches is provided to create and identify molecular nanosensors. Bottlenecks are discussed and opportunities of non‐classical recognition strategies are highlighted such as corona phase molecular recognition as well as the requirements for high throughput and scalability. Fluorescent carbon nanotubes are powerful building blocks for sensors and their huge chemical design space makes them an ideal platform for high throughput approaches. Therefore, they are the focus of this article, but the insights are transferable to any nanosensor system. Overall, this perspective aims to provide a fresh perspective to overcome current challenges in the nanosensor field.

## Graphical abstract

Nanomaterial‐based optical sensors are powerful tools to identify and image (biological) molecules. Optimizing surface chemistry and photophysics of these sensors is essential, but current methods limit throughput. This article discusses bottlenecks and highlights opportunities and requirements for high throughput and the scalability of non‐classical recognition strategies.

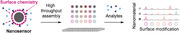

## 1 Introduction

To feed the predicted world population of 9.7 billion people in 2050, it is required to raise the food production by ≈40% between 2012 and 2050.^[^ ^1^ ^]^ To address this challenge, novel technologies such as remote monitoring of plant health are required.^[^ ^2^, ^3^, ^4^ ^]^ Moreover, the severe acute respiratory syndrome corona virus type 2 (SARS CoV‐2) pandemic demonstrated that we need rapidly adaptable sensing technologies to contain the spread of infectious diseases.^[^ ^5^ ^]^ The technological toolbox contains methods to amplify nucleic acids via polymerase chain reactions (PCR), quantify proteins via chemiluminescent or enzyme‐linked immunosorbent assays (ELISA) and detect biomarker via point‐of‐care (POC) tests.^[^ ^5^, ^6^, ^7^ ^]^ Additionally, fluorescent materials and energy transfer mechanisms can be used for molecular imaging, sensing of chemical species and non‐invasive diagnostics.^[^ ^8^, ^9^, ^10^, ^11^, ^12^, ^13^ ^]^ Nanomaterial‐based sensors are uniquely suited for such applications because many of them possess highly tunable and environmentally sensitive optoelectronic properties.^[^ ^14^ ^]^ Compared to conventional fluorophores, their emission spectra are often narrow and in desired spectral ranges such as the near infrared (NIR).^[^ ^14^, ^15^ ^]^ Moreover, these materials are typically very photostable and can be chemically tailored for sensing by modifying them with (biological) recognition units.^[^ ^16^, ^17^, ^18^ ^]^

During the last few years, the field of nanosensors has seen tremendous progress and many novel functionalization techniques have been developed.^[^ ^16^, ^19^, ^20^, ^21^ ^]^ While the large chemical space provides room for explorative work and optimization of the sensor performance, sampling the chemical space scales with the number of combinations and can become challenging. In this context, rational approaches provide one mean for efficient functionalization.^[^ ^22^ ^]^ On the other side, high throughput approaches, in which a large combinational space is sampled, promise potential for analytes that are difficult to address with traditional recognition units such as antibodies, e.g., to detect small molecules. In this article we define the lower limit for a high throughput approach as 100 variations (of sensor chemistry) but obviously the goal are numbers orders of magnitude higher.

In this article, we provide our perspective on optical nanosensor design and the challenges to efficiently create sensors with a desired performance. This optimization of nanosensors is, in our opinion, the central challenge because it determines how far sensors can be optimized, how deep our understanding of molecular recognition goes, and whether machine learning methods can be efficiently applied.^[^ ^19^ ^]^

One particularly well‐suited class of nanomaterials are single‐walled carbon nanotubes (SWCNTs). Semiconducting SWCNTs are 1D, photostable nanomaterials that fluoresce in the near‐infrared (NIR) tissue transparency region. This spectral range is highly interesting for applications in biological environments as background signals are reduced.^[^ ^23^, ^24^, ^25^ ^]^ They can be functionalized covalently^[^ ^26^, ^27^ ^]^ or non‐covalently.^[^ ^16^ ^]^ Especially non‐covalent approaches that form a novel organic corona phase present a straightforward design path to achieve high throughput. These corona phases can be adapted to almost any analyte. This type of molecular recognition by an organic phase has been coined corona phase molecular recognition (CoPhMoRe) and is compatible with many of concepts from bioengineering and synthetic biology. As many of the concepts that were developed for SWCNT‐based sensors have not been transferred to other nanosensor classes, this article highlights insights from SWCNT‐based nanosensors and general concepts that are applicable to other nanosensor classes as well.

In the first part of this perspective, we introduce how the different approaches, that are used to create nanosensors affect the scalability and develop categories (synthesis‐limited/selection‐limited). In Section 2, we discuss the main building blocks that are required for nanosensors and how they in turn affect the throughput. Section 3, provides examples that showcase progress in synthesis‐limited approaches as well as open questions and challenges for the future. In this context, we also provide a detailed summary of corona phase molecular recognition approaches and discuss how selection‐limited approaches can further increase throughput. To provide the reader with some applications of these sensors, we furthermore compiled an overview of different sensors as well as their limitations (**Table** **1**). Finally, we provide our perspective on the use of computational methods and discuss how sensor integration can be used to further fine‐tune sensors.

**Table 1:** Overview of synthesis‐limited approaches that make use of molecular recognition in the corona phase around SWCNTS (CoPhMoRe). (PVA: polyvinyl alcohol; PEG: poly(ethylene glycol), PL: phospholipid; PE: phosphoethanolamine, RAFT: reversible addition−fragmentation chain transfer (polymerization)).

Target class	Number of functionalizations	Functionalization	Screened analytes	Best functionalization	Sensitivity
Nitroaromatics^[^ ^143^ ^]^	24 phases 3 + 8 chiralities	Bombolitin II, PVA, (AT)_15_	42	Wavelength shift of Bombolitin II for different analytes. Selective response of (AT)_15_: TNT	Single molecule
Riboflavin, L‐thyroxine, oestradiol^[^ ^31^ ^]^	12	Linked rhodamines (2), PEG‐PL (1), other PEG‐derivatives (3) DNA (1) Dextrane‐derivatives (2) PVA Surfactants (2)	36 + 1 control	Riboflavin: boronic‐acid substituted phenoxydextran L‐thyroxine: Fmoc‐Phe‐PPEG8 Oestradiol: rhodamine isothiocyanate‐difunctionalized PEG	LOD not determined, range of *K*_d_’s
Neurotransmitter^[^ ^85^ ^]^	30	Nucleic acids (13), Phospholipids (12), Amphiphilic polymers (5)	9	Dopamine: (GT)_15_	LOD: 11 × 10^−9^ m *K*_d_: 433 × 10^−9^ m
Fribrinogen from proteins in blood^[^ ^29^ ^]^	20	Nucleic acids (9), PEG‐PL (11)	14	Fibrinogen:dipalmitoyl‐phosphatidylethanolamine (DPPE)‐PEG	Lowest concentration tested: 0.05 mg mL^−1^,
Insulin^[^ ^144^ ^]^	24 phases 18 + 3 chiralities	PEG‐PL	14	N‐palmitoylsphingosine‐1‐ succinyl[methoxyPEG2000]	LOD not detected 20 µg mL^−1^ (during screen) Between 180 × 10^−12^ m and 3.5 × 10^−6^ m tested
Fat soluble vitamins^[^ ^115^ ^]^	4	DNA	4	β‐Carotene: (GT)_15_	*K*_d_: 2.2 × 10^−6^ m
Phosphodiesterase type 5 inhibitor^[^ ^145^ ^]^	24	RAFT polymer	23	Vardenafil: MA‐ST‐90	LOD: 0.02 × 10^−6^ m–0.2 × 10^−6^ m
Steroids^[^ ^146^ ^]^	80 phases 16 + 5 chiralities	RAFT polymer	11	Progesterone: P10	LOD: × 10^−6^ m range
Polyphenols^[^ ^147^ ^]^	10	DNA (8), PEG‐PL (2)	10 + 1 control	Gallic acid: (C)_30_ Tannic acid: 18:0 PEG5000 PE	*K*_d_: 90 × 10^−9^ m
Auxins^[^ ^148^ ^]^	6	Polyfluorene (PF)‐, poly(4‐ vinylpyridine)‐, poly(N‐vinylimidazole)‐based copolymers	12 + 1 control	1‐naphthalene acetic acid: PVIMel 2,4‐dichlorophenoxyacetic acid: PF(1,3‐P)HCl	LOD: 8.2 × 10^−6^ m (1‐naphthalene acetic acid), 0.35 × 10^−6^ m (2,4‐dichlorophenoxyacetic acid)
Viral proteins (spike + nucleocapsid)^[^ ^149^ ^]^	11	PEG‐PL	2 (+ subsequent saliva + BSA control)	Spike: 14:0 PEG2000 PE Nucleocapsid: 18:0 PEG1000 PE	LOD 350 × 10^−12^ m (Spike), 48 × 10^−15^ m (nucleocapsid)
Onkometabolites^[^ ^150^ ^]^	9	DNA	15	D‐2‐hydroxyglutarate: (ATTT)_7_	*K*_d_: 2.66 mg mL^−1^
Divalent ions^[^ ^151^ ^]^	20	DNA	9	Cd^2+^: (A)_15_, pH 8 Co^2+^: (C)_30_, pH 8 Hg^2+^: (CCCCAT)_5_CCCC, pH 8 Cu^2+^, (C)_10_, pH8 Ni^2+^, (C)_30_, pH8 Mn^2+^, (C)_10_, pH8 Zn^2+^, (A)_15_, pH 8 Pb^2+^, (C)_15_, pH 8 Cr^2+^: TTCAATACATACGTGACCCAGTAGTTATCC, pH5.7	LOD: 33 × 10^−9^ m (Hg^2+^)
Gynecologic cancer markers^[^ ^152^ ^]^	11	DNA	3 + 22 patient samples	Identification via ML from spectral fingerprint	Classification at subnanomolar concentration
Ovarian cancer^[^ ^30^ ^]^	10	DNA + sp^3^‐defects	269 patient samples	Identification via ML from spectral fingerprint, most important sensor: 3F*(TAT)_4_	87% sensitivity at 98% specificity (compared with 84% sensitivity at 98% specificity for the current best clinical screening test
Chemotherapeutics^[^ ^153^ ^]^	10	DNA	4	Temozolomide and 5‐aminoimidazole‐4‐carboxamide: (GGGT)_3_	LOD: <30 × 10^−6^ m
Gibberellins^[^ ^154^ ^]^	11	RAFT polymer	15	GA_3_^−^K^+^: S‐gluAPM GA_4_^−^H^+^: N‐gluAPM	LOD: 542 × 10^−9^ m (GA_3_^−^K^+^), 2.96 × 10^−6^ m (GA_4_^−^H^+^)
Inflammatory cytokines^[^ ^155^ ^]^	164 phases (41 and 4 chiralities)	RAFT polymer	19	Interleukin‐6: MK2	
Enzymatic substrates/products^[^ ^106^ ^]^	10	DNA (9) PEG‐PL (1)	13 + 2 controls	*p*‐phenylene/Brandowskis base: (G_2_T)_10,_ TMB: (C)_30,_ ONPG: (GA)_15_	LOD: 2.5 × 10^−9^ m (PPD—for HRP reaction in × 10^−12^ m range)
Viral nucleocapsid proteins^[^ ^105^ ^]^	14	PEG‐PL	7	SARS: 16:0 PEG2000 PE MERS: 14:0 PEG750 PE SARSCoV‐2: 18:1 PEG5000 PE H1N1: 18:0 PEG5000 PE H3N2: 18:0 PEG2000 PE Lassa: 16:0 PEG750 PE Ebola: 14:0 PEG1000 PE	LOD: 18.9 × 10^−12^ m (SARS) 3.5 × 10^−12^ m (MERS) 2.1 × 10^−12^ m (SARS‐CoV‐2) 7.1 × 10^−12^ m (H1N1) 13.3 × 10^−12^ m (H3N2) 16.6 × 10^−12^ m (Lassa) 100.5 × 10^−12^ m (Ebola)

## 2 Scalability of Nanosensor Engineering

**Figure 1:**
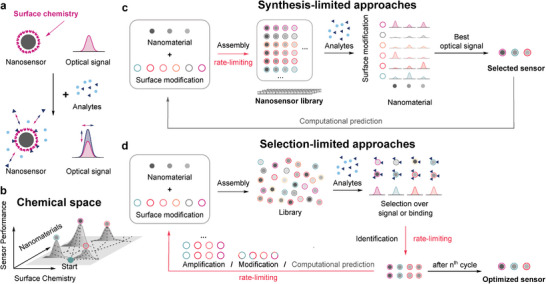
High throughput approaches to create nanosensors. a) Nanosensors translate binding of analytes into optoelectronic changes. b) The combination of different nanomaterials and surface modifications allows for an optimization of the sensor performance. While a large number of combinations provides access to different optimization paths, the costs to explore the chemical space scale in a similar way and require efficient sampling. c) In synthesis‐limited approaches, a nanosensor library is assembled, analytes are added to the known nanosensors and the response is measured, e.g., in a well‐plate format (screening). Typically, the limiting step is assembly/synthesis. d) Selection‐limited approaches isolate sensors with desirable signal change or binding behavior from a pool of sensor candidates prior to the identification of the nanosensor. In this process, the surface functionalization of a nanosensor can be selected from a large library or further modified via mutation. An iteration of this process leads to a selective enrichment of optimized sensors. Typically, the limiting step is selection/identification.

Optically active nanomaterials are versatile building blocks for sensing approaches. Compared to conventional fluorophores, nanoscale materials such as plasmonic nanoparticles, SWCNTs, and graphene possess a high photostability. As photobleaching can be excluded, these materials enable testing of many conditions and become interesting for high throughput approaches. To turn a material into a sensor, the surface is commonly modified to selectively interact with an analyte (**Figure** **1**a, see also Section 2). The optical properties as well as chemical tunability allows for different optimization paths. However, an efficient optimization can be challenging and requires efficient techniques for large‐scale sampling of the physical and chemical design space (Figure 1b).^[^ ^28^ ^]^

**Figure 2:**
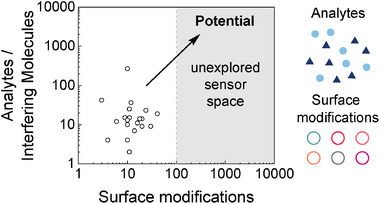
Design space and analyte numbers of SWCNT‐based sensors using synthesis‐limited approaches. In non‐classical recognition approaches, sensors with chemically distinct interphases (surface modifications) are screened against a panel of analytes and interfering molecules. So far, the approach is limited by the preparation of sensors with different surface chemistries. It represents a synthesis‐limited high throughput approach. Here, numbers from published papers using this approach are shown (summarized in Table 1).

In the simplest case, nanomaterials with different shapes, sizes, and/or compositions are separately modified with different surface modifications. Subsequently, different target analytes are added, and their optical response is monitored to select suitable combinations for further optimization (Figure 1c). Using such a format, it is possible to understand molecular recognition of different hybrid nanomaterials and identify combinations that are not selective on their own but form through their interaction with selective binding pockets.^[^ ^29^, ^30^, ^31^ ^]^ When available, it is possible to include rational design, chemical intuition, and computational predictions as guidelines for an efficient sampling. However, these principles can be difficult to apply for nanomaterials as the combinations are limitless and the interactions on the surface of a material can be complex. For example, SWCNT‐based sensors are often modified with single stranded DNA strands. When confined to a nanomaterial, DNA behaves significantly different and forms hydrogen bonds that would be not typical in solution.^[^ ^32^, ^33^ ^]^ This behavior leads to the formation of complex interfaces that are highly dynamic and can interact as a non‐classical recognition unit with analytes, in which the interaction is an emergent property of nanoparticle, the polymer (e.g., DNA) and the respective (competing) analytes. A typical 30 base long oligo nucleotide allows 4^30^ different permutations. Moreover, there are multiple oligonucleotides on a single SWCNT, e.g., ≈265 (GT)_15_‐oligonucleotides,^[^ ^34^ ^]^ which further increases the number of possible permutations to (4^30^)^265^. A small panel of such sensors with different interfaces (corona phases) can be synthesized and tested against a panel of different analytes. Most often, the bottleneck of such a high throughput approach is sensor preparation/assembly. Therefore, we propose to call this type of strategy a synthesis‐limited high throughput approach as the rate‐limiting step is a synthesis of different sensors (**Figure** **2**).

To circumvent the challenges of rational design, researchers drew inspiration from bioengineering approaches and developed methods that optimize the screening process. In contrast to synthesis‐limited methods, the sensor optimization of such selection‐limited approaches proceeds over multiple iterations in which suitable surface modifications are either enriched from a random library or actively modified in an attempt to further improve the sensors (Figure 1d).^[^ ^35^, ^36^ ^]^

We use this terminology and classification to show that they are different in terms of what one could call the bottleneck or rate‐determining step. For synthesis‐limited high throughput approaches the current bottleneck is the assembly of many different sensors. In screening‐based approaches, multiple different sensors are synthesized in one pot and create a pool of sensor candidates. This allows for a much higher number of nanosensors but it also means that one does not know the exact design of the sensors with the highest performance, affinity, etc. as many sensor combinations are present in the same sample and become enriched over multiple cycles. Therefore, the rate‐limiting step is typically identification/selection and we call it a selection‐limited approach. To allow for the identification of the sensors, the surface chemistry of this approach must fulfill synthetic conditions, e.g., primers need to be incorporated (see Section 3.2.).

Conceptualizing these two different approaches is important to understanding bottlenecks in the development of nanosensors and in general the biosensor field. It pinpoints to the steps in developing novel nanosensors that are crucial and need most attention from the field.

## 3 Potential Materials and Sensor Design

**Figure 3:**
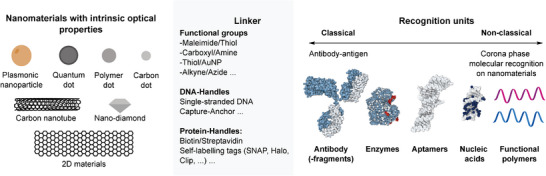
Factors affecting the scalability of nanosensor assembly. Nanosensors are typically composed of an optically sensitive nanomaterial, a recognition unit that interacts with an analyte, and a linker chemistry that connects both. Very often the linker and the recognition unit are the same and the physiochemical processes are connected. The ease of chemical modification/permutation affects the scalability.

Some materials have properties that enable scalability more easily. To create a (optical) nanosensor, optically active (e.g., fluorescent) materials are combined with recognition units that permit molecular recognition (**Figure** **3**). To link both components, a suitable linker strategy is required. As the sensor response of most nanosensors is caused by a perturbation of the nanoparticle environment (corona) upon binding, the following section will briefly introduce the main “components” as well as the main properties that affect sensing.

### 3.1 Nanomaterials

Nanoscale objects often exhibit size‐dependent properties. Of those materials, metallic nanoparticles represent, due to the high tunability of size and optical properties, the most frequently used imaging modality.^[^ ^37^, ^38^, ^39^, ^40^, ^41^ ^]^ Pure metals (Au, Cu, Ag, …) are electronically conducting and their optical properties depend on surface plasmon resonance (SPR) effects.^[^ ^42^, ^43^ ^]^ Consequently, changes in their optical properties are typically due to a change in an oscillating electric field, e.g., coupling effects between materials with different resonances.^[^ ^28^ ^]^ In contrast, quantum dots (Qdots) are metal chalcogenides (CdSe, PbS, InAs, …) with a size‐dependent band gap that determines their optical properties.^[^ ^44^, ^45^, ^46^, ^47^, ^48^ ^]^ Originally, the term “dot” was used to describe the size of particle and as a result different fluorescent nanoparticles fall under the umbrella term “dot.”^[^ ^15^, ^48^, ^49^, ^50^, ^51^, ^52^, ^53^ ^]^ Semiconducting polymer dots (Pdots), can contain non fluorescent molecules but are mainly composed of semiconducting polymers and exhibit a comparable size to Qdots.^[^ ^49^, ^54^, ^55^, ^56^, ^57^ ^]^ A smaller version, carbon‐based derivative are carbon dots. Carbon dots are spherical nanomaterials with a diameter between 1 and 10 nm. Their π‐system absorbs light in the ultraviolet UV range and emits light in the visible. Similarly to carbon dots, SWCNTs also possess inherent fluorescent properties.^[^ ^58^, ^59^ ^]^ However, semiconducting SWCNTs are fluorescent in the NIR and can be excited at different wavelengths that correspond to the respective energy transitions (Typically E_11_: NIR; E_22_: VIS; E_33_: UV). Moreover, SWCNTs are photostable and do not bleach.^[^ ^60^ ^]^ The nature of the fluorescence in SWCNTs is excitonic.^[^ ^61^ ^]^ Excitons in SWCNTs have a size on the order of nm and diffuse over the length of a few hundred nanometers.^[^ ^62^, ^63^, ^64^ ^]^ This renders the fluorescence of SWCNTs highly sensitive to their chemical surrounding, which is a key advantage to other fluorescent materials that are relatively inert. The introduction of quantum defects (sometimes called organic color centers) to SWCNTs provides an additional opportunity to tune the fluorescent properties of SWCNTs and provides a handle for further functionalization.^[^ ^26^, ^27^, ^65^, ^66^ ^]^

### 3.2 Linker

To functionalize a nanomaterial for (bio‐)sensing, recognition units need to be connected to the nanomaterial. In this context, conjugation methods with high yields are often used to ensure efficient functionalization.^[^ ^67^ ^]^ Examples include traditional chemical strategies such as reactions between maleimides and thiols, carboxyl groups and amines, the reactions between alkynes and azides (CuAAC, SPAAC) as well as the functionalization of gold nanoparticles with thiols.^[^ ^17^, ^68^, ^69^ ^]^ For an overview of different bioconjugate techniques see for example.^[^ ^47^, ^67^, ^70^, ^71^, ^72^, ^73^, ^74^, ^75^, ^76^, ^77^ ^]^ Additionally, the introduction of protein handles (biotin/streptavidin, His‐, SNAP‐, Halo‐, Clip‐tags, …) and DNA‐based approaches relying on hybridization, or the formation of selective interfaces allow for rapid assembly of nanosensors. While functionalizing a nanomaterial, it is important to remember, that the optical properties of a material can be affected by its functionalization. For example, the introduction of quantum defects with low defect densities leads to novel fluorescent features that can be used to tune the quantum yield^[^ ^78^, ^79^ ^]^ and sensing properties of SWCNTs.^[^ ^80^, ^81^, ^82^ ^]^ An extensive functionalization of a nanoparticle can, however, lead to the loss of its fluorescent properties, if the fluorescence is affected by the surface modification. Similarly, the controlled fixation of anchor‐structures on the surface of highly sensitive nanoparticles can be used as a tailored passivation strategy to improve selectivity and stability.^[^ ^22^, ^83^ ^]^ By constraining a recognition unit to such an anchor, the conformational freedom of a recognition unit can be further influenced.^[^ ^84^ ^]^ Such approaches furthermore may therefore influence the binding affinities and allow for an optimization of the signal translation and selectivity before the introduction of recognition units thereby providing an interesting opportunity for high throughput approaches.

### 3.3 Recognition Units

To render a nanomaterial sensitive to a specific analyte class, an analyte needs to interact and modify the fluorescent properties of a nanomaterial. Nanomaterials are therefore modified with recognition units that provide some selectivity toward the analyte. Classical examples of such interactions are antibodies and aptamers that are optimized to bind to a respective analyte.^[^ ^22^, ^85^, ^86^, ^87^ ^]^ Additionally, smaller fragments^[^ ^84^, ^88^ ^]^ or non‐traditional recognition units such as nucleic acids and functional polymers can be interesting because they can form selective corona phases^[^ ^24^ ^]^ on the nanomaterial that serves both recognition and signal transduction. While the attachment of an antibody (‐fragment), aptamer or nucleic acid allows for maximum rationality in the approach, the throughput is often limited by a low number of available recognition units and the relatively high cost associated with the previous optimization. Additionally, most nanomaterials are only affected by perturbations in their direct vicinity. Consequently, the precise orientation of the recognition unit can be important for the performance of a nanosensor which renders larger recognition units difficult to implement in high throughput approaches. This said, most larger screenings are based on evolving “non‐rational” corona phases.^[^ ^89^ ^]^ These corona phases contain polymers that adsorb on the nanomaterial, as well as analytes that bind to this complex interface. Consequently, an evolution of adsorption‐based surface modifications and methods combining such adsorption‐based approaches with a rational design are highly desirable.

### 3.4 Sensor Design in Biological Environments and Translation to In Vivo

Biological environments are complex and crowded when compared to simple buffer systems.^[^ ^90^ ^]^ When engineering a sensor for these systems, it becomes important to consider this aspect for the practical application of sensor materials, as well as the design of a suitable surface chemistry for these environments. For the latter, it is important to validate and document the sensor safety and the performance under clinical/biological conditions. To address safety concerns, standard guidelines and methodologies for the characterization and safety assessment of nanomaterials, such as MIRIBEL,^[^ ^91^ ^]^ are available. Depending on the sensor mechanism and the scale‐up method, it can be efficient to perform the screenings and the optimization of sensors in an environment that is close to the in vivo conditions in an application. By choosing an appropriate material, it is furthermore possible to select the appropriate properties and spectral region for a given application. Visible fluorophores, typically provide a readout modality that is easy to access with conventional instrumentation or useful for point of care settings in resource‐constrained settings (naked‐eye detection).^[^ ^92^, ^93^, ^94^ ^]^ From a chemical perspective, the light‐matter interactions in in biological samples (absorption, scattering, autofluorescence) can lead to a low signal to noise ratio (contrast) in the visible region.^[^ ^23^, ^24^, ^25^ ^]^ As the absorption, scattering and autofluorescence of biological samples is reduced and higher penetration depths can be reached in the NIR.^[^ ^95^, ^96^ ^]^ This can be an advantage for applications that require a high spatiotemporal resolution in biological samples, such as the in vivo detection of bacteria, through scull imaging of the brain, or vascular imaging.^[^ ^97^, ^98^, ^99^ ^]^ When used for imaging purposes, it is important to consider that the resolution scales with the wavelength and that the performance is dependent on the detection methodology, e.g., the sensitivity of a camera or detector in a specific spectral range and the resolution.^[^ ^100^ ^]^

## 4 High Throughput Strategies

As for all high throughput approaches, the scalability of sensor preparation and their characterization is an important factor for synthesis‐limited as well as selection‐limited approaches. In this section, we will present an overview of design considerations and conditions for high throughput screenings.

### 4.1 Synthesis‐Limited High Throughput Approaches

**Figure 4:**
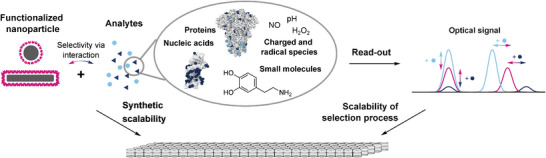
Implications of the sensor synthesis and optical readout for scalability of sensor engineering. The sensor functionalization determines the chemical interactions between nanosensors and different analytes. This interplay is responsible for the generated optical read‐outs. Both aspects affect the scalability of any high throughput strategy.

From a synthetic perspective, the design of a nanosensor library is mainly determined by the chemical properties of the nanomaterial. Since a complete overview would be beyond the scope of this perspective, interested readers are directed toward several excellent reviews.^[^ ^19^, ^101^, ^102^, ^103^ ^]^ For example, gold nanoparticles can be easily modified via thiols. In contrast, sp^2^‐hybridized particles such as SWCNTs can be modified via non‐covalent, adsorption‐based functionalization via π‐stacking and covalent functionalization via nucleophilic, electrophilic, or radical reactions. Due to their ease of functionalization and their high sensitivity toward their chemical environment (coronae), SWCNTs are interesting building blocks that are i) easy to modify chemically and ii) able to translate binding events into optical signals (**Figure** **4**). Furthermore, SWCNTs have historically been used abundantly with non‐rational design concepts that are easily scalable in screening approaches. We will therefore, discuss differences in high throughput methods by using SWCNT‐based sensors as an example.

#### 4.1.1 Serial Single Sensor Screening

**Figure 5:**
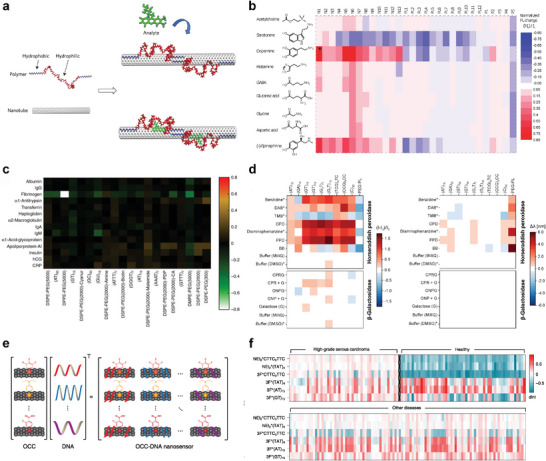
Synthesis‐limited screening approaches. a) Corona phase molecular recognition (CoPhMoRe) concept. Physisorption of a biopolymer on a nanomaterial creates a novel organic phase (corona) that is— together with the nanomaterial—capable of recognizing (bio)molecules. Reproduced with permission.^[^ ^31^ ^]^ Copyright 2013, Nature. b) Response of sensors coated with DNA (N1–N13), phospholipids (PL1‐12) and amphiphilic polymers (P1–P5) against different neurotransmitters. Reproduced with permission.^[^ ^85^ ^]^ Copyright 2014, American Chemical Society. c) Intensity changes of DNA‐ and phospholipid sensors after the addition of different proteins from blood. Reproduced with permission.^[^ ^29^ ^]^ Copyright 2016, Springer Nature. d) CoPhMoRe screen to identify sensors for enzymatic substrates and products. Note that the intensity changes of CoPhMoRe sensors (left) are independent from wavelength changes. Adapted with permission.^[^ ^106^ ^]^ Copyright 2024. The Authors. Wiley‐VCH. e) Extension of the classical CoPhMoRe concept to organic color centers (OCC). f) Response of OCC‐/DNA‐sensors to 215 serum samples from individuals with high‐grade serous ovarian carcinoma and other diseases. Note that this sensor array outperformed the best clinical screening test for this disease. e,f) Reproduced with permission.^[^ ^30^ ^]^ Copyright 2022, Nature.

SWCNT‐based sensors can be generated via rational design, screening methods, or selection methods. While rational design allows for a maximum degree of control and more scalable methods have been developed recently,^[^ ^22^, ^65^, ^104^ ^]^ there is up to our knowledge no example of a rational bottom‐up assembly with covalently functionalized SWCNTs that would allow to be scaled towards a high throughput approach. Instead, SWCNTs are in the context of screenings most often functionalized non‐covalently via the adsorption of different polymers. When confined to the surface of a SWCNT, these polymers can form complex interfaces that act as a universal receptor which that selectively bind the analyte. (**Figure** **5**a).

Compared to other methods, this corona phase molecular recognition (CoPhMoRe, Figure 5b–d) can be used to generate sensors to hypothetically any analyte (Table 1). The achievable selectivity should scale with the library and size, which highlights again why scalability is a central question for biosensor engineering. In most examples, 10–40 interfaces are tested against a library of approx. 5–50 analytes (Table 1, Figure 2). These numbers can be explained by the serial preparation of such nanosensors that include pipetting, sonication, functionalization and centrifugation steps that are difficult to upscale.

Often, the thereby generated sensors are sensitive in the µM–nM range. However, depending on the optical set‐up, sample volume, and functionalization approach, the generation of sensors with sensitivities in the pM–fM are feasible. To investigate the influence of functional groups on the surface coverage, hydrodynamic radius, and the interactions leading to molecular recognition of an analyte, it is possible to simulate the 3D corona phase with molecular dynamics and docking experiments.^[^ ^105^ ^]^

While CoPhMoRe promises to be a universally tunable recognition sequence, one main bottleneck of using highly sensitive nanomaterials are small perturbations (from unspecific binding) that result in high signal changes. To circumvent this problem, it is possible to i) tailor the rigidity of the soft interfaces at the nanosensor surface^[^ ^22^, ^83^ ^]^ or ii) use multiple nanosensors in a multiplexed format.^[^ ^107^ ^]^ It is possible to combine covalent surface modifications (sp^3^‐defects/OCC: organic color centers) and non‐covalently adsorbed polymers (Figure 5e,f) to expand the chemical space.^[^ ^30^ ^]^ Additionally, different linking strategies can be combined and the recognition unit can be constrained to bridging anchor molecules on the surface of a SWCNT.^[^ ^84^ ^]^

#### 4.1.2 Applications with Array‐Based Screening

The optimization of sensors presents a key step for the generation of nanosensors and when one functionalization provides sufficient selectivity, a simple coating of one nanosensor type on a surface presents a straightforward approach.^[^ ^29^, ^108^, ^109^, ^110^, ^111^, ^112^ ^]^ However, using just one sensor may be of limited use in complex chemical environments and the translation into an industrial or clinical setting. Here, ratiometric sensing approaches can improve the accuracy of a sensor by compensating for variations in mechanical movements and variations of excitation intensities.^[^ ^113^ ^]^ Further, multiplexing of sensors presents interesting opportunities for sensing different analytes in complex environments.^[^ ^107^, ^114^ ^]^

**Figure 6:**
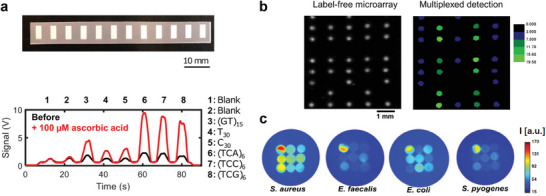
Multiplexing of sensors presents opportunities for rapid signal acquisition of multiple sensors and improved selectivity. a) Sensor barcode of a paper‐based barcode with differently functionalized SWCNTs fixed on a plastic strip (top). Measurement of the barcode before and after dipping the barcode in ascorbic acid. Reproduced with permission.^[^ ^115^ ^]^ Copyright 2020 American Chemical Society. b) Multiplexed microarray for real time detection of mouse IgM. SWCNTs are modified with chitosan polymers and further functionalized via chelation between NTA, Cu^2+^ ions, and His‐tagged capture proteins. Binding to the capture protein modifies the distance of Cu^2+^ to the SWCNT which serves as proximity quenchers of the SWCNT fluorescence. Reproduced with permission.^[^ ^116^ ^]^ Copyright 2018, American Chemical Society. c) Optical identification of pathogens with a SWCNT‐sensor array. Different nanosensors are incorporated in a hydrogel, their multiplexed response allows the differentiation of pathogenic bacteria. Adapted under the terms of a CC‐BY 4.0. license.^[^ ^107^ ^]^ Copyright 2020, The Authors.

As a lightweight and inexpensive substrate, paper has been widely used for sensing approaches. By using a wax printing method, it was possible to design custom SWCNT‐based barcodes on paper substrates, that can be fixed on other substrates (**Figure** **6**a).^[^ ^115^ ^]^ Additionally, microarrays can be printed on substrates such as glass. For example, the deposition of nanoliter volumes leads to spot sizes in the order of approx. 210 µm with spot heights of approx. 50 nm (Figure 6b).^[^ ^116^ ^]^ Interestingly, the sensitivity of the nanotubes is often not limited by the SWCNT chemistry but by the detector size needed to accommodate the image. Therefore, using a higher imaging magnification can sometimes increase the detection limit by one order of magnitude at the expense of a smaller field of view.^[^ ^116^ ^]^ A third option is the incorporation of multiple different sensors into a hydrogel or another type of polymer (Figure 6c).^[^ ^107^ ^]^ This approach is very useful in complex mixtures to identify bacteria based on their chemical fingerprint, which is crucial for biomedical diagnostics. Here, the hydrogel can also serve as another design parameter for example by affecting the diffusion of the analytes to the sensors.

Overall, the main conceptual advantage of array‐based techniques is the parallel analysis of multiple sensor responses to a single analyte by imaging instead of serial screening the responses.

### 4.2 Selection‐Limited High Throughput Methods

Compared to synthesis‐limited approaches, which are inherently restricted in their throughput, the iterative optimization of surface functionalization allows for a larger exploration of chemical space. To scale the throughput efficiently, the surface of nanomaterials is modified with a library of different polymers. Subsequently, (bio)polymers (so far DNA) that contribute to the selectivity of the sensor are either i) amplified over multiple selection rounds, or ii) based on their performance selected and subsequently modified to optimize sensors.

#### 4.2.1 Systematic Evolution of Ligands on Nanomaterials (SELEC)

**Figure 7:**
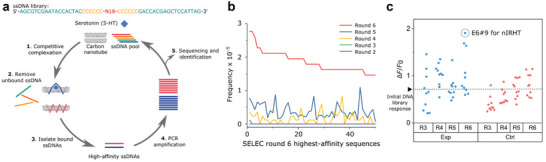
Systematic evolution of ligands on nanomaterials (SELEC). a) Overview of the selection process. In contrast to directed evolution, the same DNA pool is used over multiple rounds, and sequences are not modified. b) Frequency of the final 50 sequences with the highest affinity during different selection rounds. (c) Increased sensor sensitivity of the experimental (selection with serotonin) and the control (selection without serotonin) SELEC groups during the selection rounds 3–6. Reproduced under terms of a CC‐BY‐NC 4.0 license.^[^ ^35^ ^]^ Copyright 2019, The Authors.

In this process, a nanomaterial is coated with an analyte and a library of polymers (**Figure** **7**a). Next, non‐binding polymers are first washed away, and subsequently, higher affinity polymers are removed from the nanomaterial surface, amplified, and used in the next selection round. Over an iteration of multiple rounds (Figure 7b), it is possible to select high‐affinity binders from libraries with up to 10^10^ polymers. When comparing the sensor responses of sequences selected in the presence and absence of an analyte, Jeong et al.^[^ ^35^ ^]^ found that those sequences that were selected in the presence of the analyte exhibited better sensor responses (Figure 7c).

Overall, SELEC assumes that the abundance of a sequence over multiple rounds is a good predictor for sensor sensitivity and selectivity. To allow for rapid amplification, these selections have to be (so far) nucleic acid based, and the sequences are amplified via polymerase chain reactions (PCR). This requires modification of the nucleotides with flanking regions (e.g., (C)_6_) and primer. As a consequence, the varied region can be, compared to the length of the amplification construct relatively short. Since nucleic acid conformations on nanoparticles are known to be dependent on self‐interaction,^[^ ^117^, ^118^ ^]^ and the variation of a few bases has been shown to alter the sensor response significantly, the conformation of the varied region is likely affected by the amplification regions as well as the large diversity of different nucleic acids in the DNA pool. Additionally, the preparation via tip sonication with the analyte as well as the separation from high‐affinity DNA imposes certain size and stability requirements on the analytes. Nevertheless, this method provides up to our knowledge the highest scalability for the identification of high affinity binders from a large pool of (bio)polymers. Conceptually, SELEC is closely related to the systematic enrichment of ligands by exponential enrichment (SELEX), the standard methodology for the generation of aptamers. Therefore, one alternative to SELEC approaches is the use of a preselected aptamer, which has also been used to create sensitive and selective sensors for serotonin.^[^ ^119^ ^]^

#### 4.2.2 Directed Evolution

**Figure 8:**
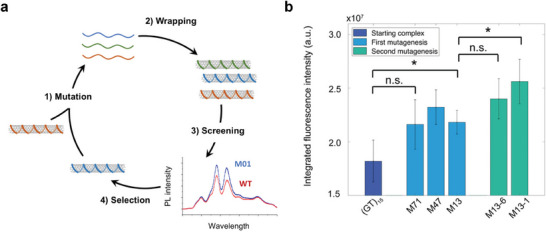
Directed evolution of nanosensors. a) Overview of the selection process. While the evolution cycle can be repeated over multiple rounds, the material is typically not used again. b) Normalized increase of the fluorescence intensity over multiple evolution rounds. Reproduced under terms of a CC‐BY‐NC 3.0. license.^[^ ^36^ ^]^ Copyright 2024, the Authors.

Originally used in protein engineering, directed evolution is a process that is used to optimize the function of a protein by successive generations of random mutations and artificial selection or screening.^[^ ^120^ ^]^ The concept is centered around the premise that the function of a protein can be tuned by modifying amino acids in the gene that is responsible for the desired protein functionality.^[^ ^121^ ^]^ Lambert et al.^[^ ^36^ ^]^ adapted this concept to the engineering of the optoelectronic properties of nanomaterials (**Figure** **8**a). To optimize the fluorescence intensity of sensors without changing their selectivity, they i) randomly mutated (GT)_15_, a commonly used oligonucleotide for neurotransmitter detection, prepared ii) the respective oligonucleotides, iii) screened and iv) choose novel sequences for the next round based on the sensors with enhanced fluorescence intensities. In the first round, the library consisted of 99 mutants and led to three mutants with enhanced properties (Figure 8b). In the second round, the best two mutants were further mutated (library of 10 mutants) to produce an additional two mutants with enhanced brightness. Importantly, the fluorescence response to dopamine was not changed compared to the original sequence and interestingly, it could be shown that a change of only three mutations leads to a change in the nanosensors optoelectronic properties.^[^ ^28^, ^36^ ^]^ More recently, Lambert et al. used a library of SWCNT‐DNA hybrids to design sensors for aflatoxin and fumonisin.^[^ ^122^ ^]^ They used directed evolution approaches to further enhance the sensor response by more than three‐fold. Additionally, they showed that DNA shuffling can be used to accelerate sensor optimization. For this, they (computationally) cut well performing sequences into smaller fragments, shuffled them, and used the sequence to prepare novel sensors. Compared to the introduction of single mutations, this approach allows to evolve the hybrid from one local minimum toward a new local optimum with higher performance. Using combinational shuffling, it is therefore possible to sampling larger chemical landscapes with a minimal extension of the library size.^[^ ^122^ ^]^ These approaches have so far a smaller throughput similar to the synthesis‐limited approaches but novel assembly procedures and multiple evolution rounds can improve the numbers.

### 4.3 Computational Tools for Selection

**Figure 9:**
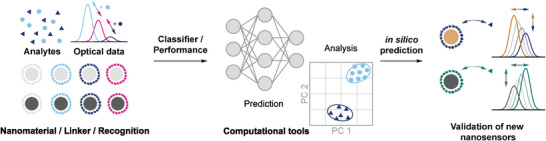
Computational approaches for the generation and analysis of nanosensors. Computational methods can be used to guide and accelerate high throughput approaches. Examples include methods for prediction and analysis of sequence patterns and sensing responses to deduce guidelines for sensor design.

Despite the success of screening methods, the synthesis of novel nanosensors presents a major bottleneck for the scalability of high throughput approaches. To reduce the time needed for the exploration of chemical space, different groups developed in silico approaches for the prediction of suitable nanosensor modifications (**Figure** **9**). Apart from prediction, computational methods can furthermore be used for classification and clustering of sensor data.^[^ ^114^, ^123^ ^]^

#### 4.3.1 Prediction of Optimal Nanosensor Functionalization

Due to the structure–function relationship of molecular recognition, the shape of a corona phase is strongly correlated with the conformation of a polymer on the nanomaterial.^[^ ^124^, ^125^ ^]^ For SWCNTs, it is known that certain sequences display high affinities to certain chiralities and can be used to separate them.^[^ ^126^, ^127^, ^128^ ^]^ Finding such selective sequences is similar to the generation of a biosensor and early studies designed to systematically sample a DNA library for chirality dependent recognition via sequence pattern achieved a success rate between 7%^[^ ^127^ ^]^ and approx. 10%.^[^ ^129^, ^130^ ^]^ To predict suitable surface modifications for biosensors, Yang et al.^[^ ^129^ ^]^ reported the first machine learning approach to molecular recognition of DNA‐SWCNTs. Using this model, the frequency of finding correct recognition sequences for the recognition of SWCNT chiralities increased above 50%. More recently, Lee et al.^[^ ^131^ ^]^ presented how machine learning could distinguish high affinity ssDNA sequences and used molecular dynamics simulations to identify patterns of intramolecular hydrogen bonding in these sequences.

Motivated by the early approaches of Yang et al., Gong et al.^[^ ^132^ ^]^ generated a DNA‐SWCNT library consisting of 1408 elements and leveraged machine learning to understand molecular recognition and sensor responses of different analytes on nanoparticles. Due to the high sensitivity of nanotubes to their chemical environment, their sensor response is strongly dependent on solution and experimental conditions. To ensure comparability, they defined the pH, ionic strength of the butter, the excitation flux, SWCNT concentration (to reduce aggregation), and time points (kinetics of sensor response) and generated data from a set of 176 sequences and a combination of two pH and four analytes. Using this data, local structure predictions were generated from a convolutional neural network (CNN) that correlated photophysical responses with a prediction of shorter‐length DNA motifs. The CNN model was, in combination with 40 high‐level features (HLF, e.g. melting point, dimers, molecular weight, etc.), used as independent features in a gradient‐boosted decision tree (GBDT). The HLF was based on a vectorization via principal component analysis (PCA). For 6 out of 8 samples, the prediction from the GBDT showed a significant correlation to the actual sensor response. The implementation of HLF improved the prediction but was found to be uniquely correlated to different analytes. Additionally, the authors found that i) different pH conditions lead to different photophysical responses, ii) the stability of the corona phases influences the sensor (more positive sensor response with decreased DNA length, increased adenine content, decreased cytosine content), iii) intensity and shape sensor responses provide orthogonal information, iv) strand‐strand interactions play a major role in the organization of the corona phase and v) molecular recognition is mechanistically different for different analytes and experimental conditions.

**Figure 10:**
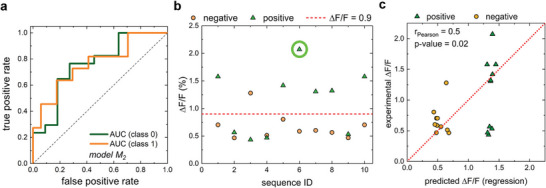
Prediction of serotonin nanosensor responses with machine learning methods. a) ROC curve of the performance of a convolutional neural network (CNN) model trained with an extended data set of 113 sequences to classify the performance of sensors as Δ*F*/*F* < 0.9: class 0 or Δ*F*/*F* > 0.9: Class 1. b) Prediction of 20 DNA‐SWCNT sensors and their responses to serotonin. The prediction is based on classification and regression models. c) Comparison of predicted and experimentally measured fluorescence changes for the sequences in (b). Reproduced with permission.^[^ ^133^ ^]^ Copyright 2022, American Chemical Society.

Based on the premise that a high performing sequence is likely obtained from a set of completely randomized sequences, Kelich et al. used a SELEC approach to narrow down a DNA‐pool of ≈10^10^ DNA‐sequences down to 100 sequences and train CNN models with single‐wavelength data from these sensors.^[^ ^133^ ^]^ The authors showed that machine learning methods can classify (**Figure** **10**a) and predict (Figure 10b,c) promising DNA sequences. Additionally, the authors demonstrated separately, that a simpler approach via a principle component analysis (PCA) appears to be predictive in analysis plots, but exhibits a poor correlation between predictions and validation experiments. Overall, the authors identified five serotonin sensors with higher responses than those previously identified in experimental screening approaches.

Most recently, Kelich et al.^[^ ^134^ ^]^ showed that the incorporation of entire spectra enhances prediction robustness and facilitates the prediction of novel sensors.

Additionally, Rabbani et al.^[^ ^135^ ^]^ and An et al.^[^ ^136^ ^]^ demonstrated a combination of machine learning and directed evolution. Rabbani et al.^[^ ^135^ ^]^ showed, that an ensemble of different models can be used to further optimize sequences that were optimized via directed evolution for mycotoxin by 5 to 40%. In contrast, An et al.^[^ ^136^ ^]^ showed a combination of directed evolution and machine learning to optimize nanosensors with improved sensitivity of serotonin over dopamine. Therefore, computational tools are very effective in navigating the huge chemical design space and proposing novel chemistries. In a certain way they are also linked to the high throughput capability of the experimental approaches because they are most effective with huge training data set.

## 5 Conclusion 

Due to their size‐dependent properties, nanomaterials can be tailored to very diverse applications. Although promising, a major challenge is the effective implementation of high throughput applications to fine‐tune sensitivity and selectivity against a panel of analytes. Herein, we provided our perspective on the current status of high throughput applications for nanosensing. Among the different materials, SWCNTs are particularly well‐suited for synthesis‐limited and selection‐limited high throughput applications. However, the main bottleneck to scale up towards high throughput approaches remains their preparation and especially surface chemistry approaches that are compatible with upscaling. Here, selection‐limited and computational approaches provide interesting opportunities to explore, analyze and predict large libraries. While larger throughputs increase the chance of finding suitable sensor properties, combinations of sensors can be used to enhance sensing by multiplexing.

Studies that shed light on the mechanism of recognition and photophysical signal transduction are crucial for an efficient design of high throughput screening and selection approaches. For example, conformational changes of DNA have been linked to fluorescence changes of dopamine nanosensors^[^ ^137^ ^]^ while redox processes have been ruled out.^[^ ^138^ ^]^ Recently, it was also shown with combined THz and fluorescence spectroscopy that local hydration changes are the key for engineering optimal sensor responses.^[^ ^139^ ^]^ Such insights refine the design considerations of the high throughput‐based approaches discussed in this perspective. However, one should not see them as competing approaches but rather complementary that benefit from each other and can inform efficient decisions for the experimental design.

The motivation and path to high throughput by novel chemistries as well as miniaturizing arrays offer huge potential not only for sensor engineering but also multiplexing and related fields such as electronic sensing, nanoparticle trafficking or gene silencing.^[^ ^21^, ^140^, ^141^, ^142^ ^]^ In summary, we anticipate that these developments open novel possibilities for the most challenging biosensing applications.

## 6 Key Definitions

### 6.1 High Throughput Approach

Approach to engineer or test many variations of nanosensors (design space and analytes). The current approaches are often limited by the efforts for synthesis (typically 10–40 sensors for a panel of 5–50 analytes) or selection (sensors are selected over multiple rounds from libraries with up to 10^10^ combinations).

### 6.2 Synthesis‐Limited Approach

Approach for the generation of nanosensors in which the limiting step is assembly/synthesis. Typically, a nanosensor library is assembled, analytes are added to the known nanosensors and the response is measured, e.g., in a well‐plate format (“screening”).

### 6.3 Selection‐Limited Approach

Approach for the generation of sensors in which the limiting step is selection/identification. Typically, selection‐limited approaches isolate sensors with desirable signal change or binding behavior from a pool of sensor candidates prior to the identification of the nanosensor. In this process, the surface functionalization of a nanosensor can be selected from a large library or further modified via mutation. An iteration of this process leads to a selective enrichment of optimized sensors.

### 6.4 Rate‐Determining Step

Step in the sensor synthesis that determines the ease of scale‐up and/or presents the biggest bottle neck. Here, it is used in analogy to the rate determining step in a chemical reaction.

### 6.5 Nanosensor

Nanoscale material that converts a chemical interaction into a signal that can be detected. Typically composed of a nanomaterial, a recognition unit and a linker.

### 6.6 Recognition Unit

A chemical or biological entity that is responsible for the interaction of (biological) analytes with the nanomaterial.

### 6.7 Corona Phase

Environment around a nanoparticle. Confinement of (bio)polymers on the surface of a nanoparticle leads to selective binding sites for analytes (corona phase molecular recognition, CoPhMoRe).

## Conflict of Interest

The authors declare no conflict of interest.
